# CAGEcleaner: reducing genomic redundancy in gene cluster mining

**DOI:** 10.1093/bioinformatics/btaf373

**Published:** 2025-06-25

**Authors:** Lucas De Vrieze, Miguel Biltjes, Sofya Lukashevich, Kodai Tsurumi, Joleen Masschelein

**Affiliations:** Department of Biology, KU Leuven, Heverlee, 3001, Belgium; Laboratory for Biomolecular Discovery & Engineering, VIB-KU Leuven Center for Microbiology, Heverlee, 3001, Belgium; Department of Biology, KU Leuven, Heverlee, 3001, Belgium; Laboratory for Biomolecular Discovery & Engineering, VIB-KU Leuven Center for Microbiology, Heverlee, 3001, Belgium; Department of Biology, KU Leuven, Heverlee, 3001, Belgium; Laboratory for Biomolecular Discovery & Engineering, VIB-KU Leuven Center for Microbiology, Heverlee, 3001, Belgium; Department of Biology, KU Leuven, Heverlee, 3001, Belgium; Laboratory for Biomolecular Discovery & Engineering, VIB-KU Leuven Center for Microbiology, Heverlee, 3001, Belgium; Department of Biology, KU Leuven, Heverlee, 3001, Belgium; Laboratory for Biomolecular Discovery & Engineering, VIB-KU Leuven Center for Microbiology, Heverlee, 3001, Belgium

## Abstract

**Summary:**

Mining homologous biosynthetic gene clusters (BGCs) typically involves searching colocalised genes against large genomic databases. However, the high degree of genomic redundancy in these databases often propagates into the resulting hit sets, complicating downstream analyses and visualization. To address this challenge, we present CAGEcleaner, a Python-based pipeline with auxiliary bash scripts designed to reduce redundancy in gene cluster hit sets by dereplicating the genomes that host these hits. CAGEcleaner integrates seamlessly with widely used gene cluster mining tools, such as cblaster and CAGECAT, enabling efficient filtering and streamlining BGC discovery workflows.

**Availability and implementation:**

Source code and documentation is hosted at GitHub (https://github.com/LucoDevro/CAGEcleaner) and Zenodo (https://doi.org/10.5281/zenodo.14726119) under an MIT license. For accessibility, CAGEcleaner is installable from Bioconda (https://anaconda.org/bioconda/cagecleaner) and PyPi (https://pypi.org/project/cagecleaner/), and is also available as a Docker image from DockerHub (https://hub.docker.com/r/lucodevro/cagecleaner).

## 1 Introduction

Biosynthetic pathways are often driven by multiple genes that are physically grouped together in the genome. Across all kingdoms of life, such gene clusters are extensively studied for their ability to direct the biosynthesis of diverse metabolites and proteins in a streamlined fashion. They play a central role in various biological processes, such as secondary metabolism, virulence, toxin production and drug resistance. Comparative analysis of gene clusters provides valuable insights into their evolutionary trajectories, and the functional and biosynthetic diversity of the pathways they encode. Tools such as MultiGeneBlast (Medema *et al.* 2013), antiSMASH (Blin *et al.* 2025) and, most recently, cblaster (Gilchrist *et al.* 2021), CAGECAT (van den Belt *et al.* 2023), zol-fai (Salamzade *et al.* 2025), and GATOR-GC (Cediel-Becerra *et al.* 2025) have facilitated such large-scale comparative analyses. These tools can provide a wide view on gene cluster diversity by querying large public genome databases, such as those hosted by NCBI. However, these large databases contain substantial genomic redundancy due to the deposition of (re)sequenced (quasi-)identical genomes, as well as from continuous sequencing efforts in the context of pathogen surveillance. This redundancy tends to propagate into the output of gene cluster mining tools, often yielding hundreds of quasi-identical clusters. As a result, a tedious curation process is typically required before meaningful downstream analyses and visualizations can be performed.

Here, we present CAGEcleaner, a Python-based pipeline with auxiliary bash scripts that rapidly dereplicates gene cluster sets by assessing the genomic similarity of their host genomes. To counteract the potential loss of gene cluster diversity caused by collapsing similar genomes, it can also recover previously discarded hits if justified by sufficient gene cluster diversity. Designed primarily as a post-processing tool for cblaster, CAGEcleaner serves as an intermediate filtering step, streamlining downstream analyses and visualizations, including those facilitated by cblaster’s sister package clinker (Gilchrist and Chooi 2021).

## 2 Implementation

**Figure 1.**
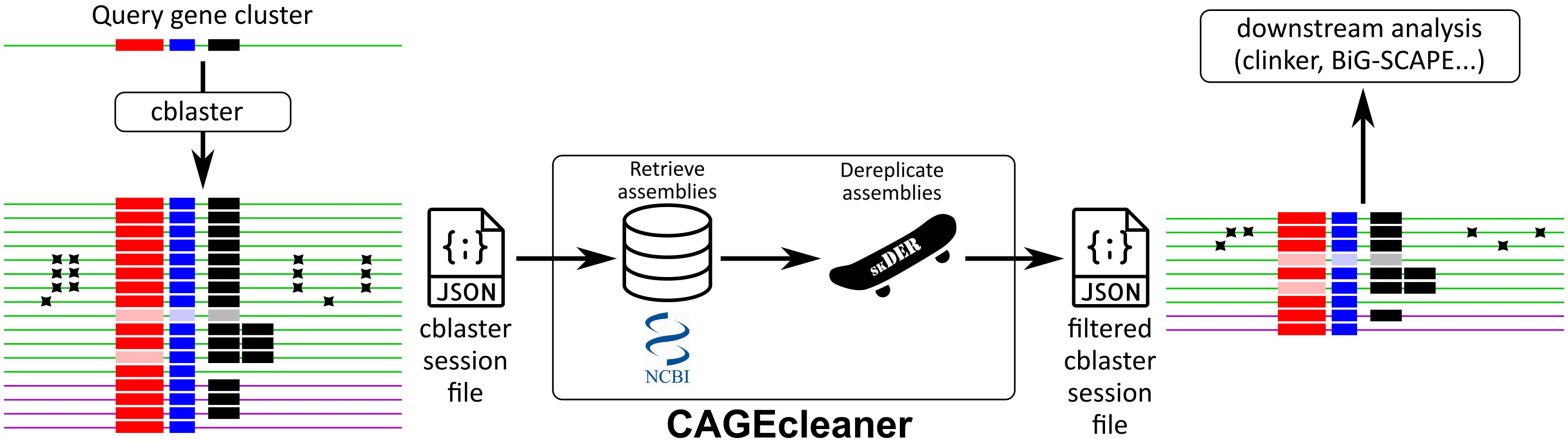
Schematic overview of the CAGEcleaner pipeline. A cblaster search query of three colocalised genes (three rectangles of different size) returned several redundant cluster hits. Some of these are from the same species as the query (upper group of lines), while other hits are from another species (lower group of lines). Some of the same-species hits are hosted by strains that are only remotely related to the query strain, which, e.g. shows in the presence of point mutations (crosses). Starting from the session file of this search, CAGEcleaner dereplicates the genomes that host these hits, and returns a filtered session file that can readily be used for downstream analyses and visualizations. It preserves some degree of genomic redundancy when justified by (i) outlier cblaster homology scores (hits with transparent rectangles), or (ii) a different number of query gene homologs than in the query cluster (hits with two or none identical rectangles).

The CAGEcleaner workflow is outlined in Fig. 1 and consists of three parts. As input, CAGEcleaner requires a cblaster session file in json-format, obtained after running a search query.

In the first part, the genome assemblies associated with the cblaster hits are retrieved. The scaffold NCBI Nucleotide IDs are first extracted from the cblaster session file and mapped to NCBI Assembly IDs using the Entrez-Direct utilities, executed via an auxiliary bash script. Scaffold entries that are part of an NCBI Whole Genome Shotgun (WGS) project are first redirected to their respective WGS master records before being mapped to Assembly IDs. The mapped assemblies are then downloaded as gzipped nucleotide FASTA files using the NCBI Datasets CLI (O’Leary *et al.* 2024) by another auxiliary bash script. To speed up ID mapping, we make the Entrez-Direct utilities process large batches of 5000 scaffold IDs at once. However, the Entrez-Direct utilities do not preserve the mapping between scaffold IDs and assembly IDs when mapping in batches. Since maintaining this mapping is critical for pinpointing scaffolds hosting gene cluster hits retained after genome dereplication, CAGEcleaner reconstructs this mapping locally by matching each scaffold ID extracted from the cblaster session file to a scaffold ID contained within one of the retrieved assembly FASTA files.

The second part of the workflow involves the dereplication of the genome assemblies. This process is performed using skDER, a fast genome dereplication tool which clusters genome assemblies based on pairwise average nucleotide identity (ANI), aligned fraction (AF), assembly N50 and connectedness metrics calculated using skani, and then selects a representative genome for each genome cluster (Salamzade and Kalan 2023). Skani is a fast genome sketching and comparison algorithm that is robust to both assembly quality (completeness, contamination, fragmentation) and sequence origin (bacterial or non-bacterial). Its performance only breaks down when analysing highly divergent genome sets (ANI < 82%) (Shaw and Yu 2023). In the CAGEcleaner workflow, skDER is run in an auxiliary bash script in greedy mode for the locally downloaded genome assemblies using a user-defined ANI threshold. Of the two modes that skDER offers, the greedy mode is more conservative and samples the genomic diversity more comprehensively than the dynamic mode. It generates smaller, more cohesive genome clusters and retains a larger number of representative genomes (Salamzade and Kalan 2023). CAGEcleaner then parses the resulting skDER output tables to identify the representative genome assemblies.

The third part of the workflow identifies the gene cluster hits to be retained by mapping the IDs of the representative genome assemblies back to gene cluster scaffold IDs using the earlier reconstructed scaffold-assembly ID mapping. In addition, it recovers gene clusters within redundant, non-representative genome assemblies that exhibit sufficient cluster diversity to justify their retention in the final gene cluster hit set. This ensures the preservation of gene cluster diversity within highly similar genomes, which may have arisen through recent genomic reorganization or horizontal gene transfer. Such hits are detected using two strategies. The first strategy evaluates the contents of each gene cluster by subdividing each skDER-identified genome cluster into subgroups based on the number of homologs for each gene in the query cluster. A new representative genome is then randomly selected from each subgroup and retained in the hit set, skipping the subgroup including the earlier retained skDER representative. However, it should be noted that this strategy does not recognize diversity in cluster contents arising from non-biological causes, such as cluster incompleteness due to highly fragmented or incomplete assemblies. Hence, such low-quality assemblies might hamper an effective dereplication when this hit recovery strategy is used. The second strategy assesses the cblaster homology scores of each gene cluster hit. Within each subgroup, hits with outlier scores as identified using z-scoring, are retained, ensuring that functionally distinct gene clusters are not removed.

Finally, CAGEcleaner generates seven output text files summarizing the dereplicated gene cluster hit sets. Intermediate output, such as downloaded genomes or skDER results, can also be returned upon request. These seven output text files are described in Table 1.

**Table 1.** Description of the seven CAGEcleaner output text files.

File	Description
clusters.txt	Comma-separated text file containing the cblaster cluster numbers of the retained gene cluster hits
filtered_binary.txt	Filtered cblaster binary presence/absence table
filtered_session.json	Filtered cblaster session file
filtered_summary.txt	Filtered cblaster summary file
genome_cluster_sizes.txt	Number of assemblies in each skDER assembly cluster
genome_cluster_status.txt	Status table with scaffold IDs, skDER representative assemblies and dereplication statuses
scaffold_assembly_pairs.txt	Internally used mapping table of scaffold IDs and assembly IDs

## 3 Example cases

We evaluated the running time, disk usage and RAM usage of CAGEcleaner for two concrete example cases. In each case, we executed the workflow using 20 CPU cores (Intel Core i7-13700k) and provided 32 GB of RAM.

In case 1, we queried four genes—the two core biosynthetic genes and their direct neighbours—from the actinorhodin biosynthetic gene cluster from *Streptomyces coelicolor* A3(2) [MIBiG (Zdouc *et al*. 2025) entry BGC0000194] against the NCBI RefSeq Protein database using cblaster at default settings. This yielded 8934 gene cluster hits in the binary table. After running CAGEcleaner at its default settings (ANI threshold of 99%), this hit set was reduced to 4847 hits, representing a 1.84-fold reduction. Among the retained hits, 170 were recovered by cluster content and 11 by outlier score. This run required 1 h 29 min, requiring 28.5 GB of disk space and 27.6 GB of memory.

In case 2, we aimed to have more redundancy in the hit set. We performed a generic query of three colocalised *Staphylococcus* genes against NCBI RefSeq Protein using cblaster at default settings and applied an Entrez query filter ‘Staphylococcus[orgn]’. This yielded 1146 gene cluster hits., which CAGEcleaner reduced to just 22 hits, a 52-fold reduction. The run required 10 min, 1.2 GB of disk space and 1.7 GB of memory.

The inputs and outputs for these two example cases are provided as Supplementary Material, available as supplementary data at *Bioinformatics* online.

## 4 Discussion

Hit redundancy is a persisting challenge in genome mining, often complicating visualization and downstream analyses. To address this issue, some of the most recent genome mining tools already implement a form of hit dereplication, e.g. the duotool zol-fai and GATOR-GC. These tools dereplicate genome mining hits at the gene cluster level and consequently do not account for the broader evolutionary context as reflected in the overall genome. In contrast, CAGEcleaner is the first pipeline to adopt a genome level dereplication approach that can discern different host species and/or strains, and preserve this genomic evolutionary signal throughout the dereplication process. This genome level dereplication strategy offers a more holistic view, but it comes with the potential drawback of inadvertently discarding unique or divergent gene clusters associated with closely related genomes. To address this, CAGEcleaner includes a hit recovery module that rescues such clusters when sufficient gene cluster diversity is detected, thereby minimizing the loss of biologically meaningful variation.

Importantly, CAGEcleaner’s genome level clustering strategy is complementary to the existing gene cluster level dereplication methods. For example, a genome harbouring a horizontally acquired gene cluster will likely cluster with the genomes of its close relatives, whereas the gene cluster itself will group together with homologous gene clusters from relatives of the original donor strain. Another example is a gene cluster that evolves at a particularly higher rate than the overall genome. The several homologs of such a gene cluster are less likely to cluster together than their host genomes would do because of this difference in evolution rate, underscoring the value of combining both genome- and cluster-level perspectives in dereplication workflows.

CAGEcleaner is implemented in Python 3 and bash. It can be freely installed from GitHub (https://github.com/LucoDevro/CAGEcleaner), Bioconda (https://anaconda.org/bioconda/cagecleaner), and PyPI (https://pypi.org/project/cagecleaner/). A Docker image is also available at DockerHub (https://hub.docker.com/r/lucodevro/cagecleaner). Source code and documentation are available on the GitHub page.

## Supplementary Material

btaf373_Supplementary_Data
